# A pocket guide to electronic laboratory notebooks in the academic life sciences

**DOI:** 10.12688/f1000research.7628.1

**Published:** 2016-01-04

**Authors:** Ulrich Dirnagl, Ingo Przesdzing

**Affiliations:** 1 Department of Experimental Neurology and Center for Stroke Research Berlin (CSB), Charité Universitätsmedizin Berlin, Berlin, 10117, Germany; 2 German Center for Neurodegenerative Diseases, Berlin, 10117, Germany; 3 German Center for Cardiovascular Diseases (DZHK), Berlin, 10117, Germany; 4 Excellence Cluster NeuroCure, Berlin, 10117, Germany; 5 Berlin Institute of Health, Berlin, 10117, Germany

**Keywords:** Code of Federal Regulations Title 21, Documentation, Data storage, Good Scientific Practice, Good Laboratory Practice, Laboratory information management systems, Software

## Abstract

Every professional doing active research in the life sciences is required to keep a laboratory notebook. However, while science has changed dramatically over the last centuries, laboratory notebooks have remained essentially unchanged since pre-modern science. We argue that the implementation of electronic laboratory notebooks (eLN) in academic research is overdue, and we provide researchers and their institutions with the background and practical knowledge to select and initiate the implementation of an eLN in their laboratories. In addition, we present data from surveying biomedical researchers and technicians regarding which hypothetical features and functionalities they hope to see implemented in an eLN, and which ones they regard as less important. We also present data on acceptance and satisfaction of those who have recently switched from paper laboratory notebook to an eLN.  We thus provide answers to the following questions: What does an electronic laboratory notebook afford a biomedical researcher, what does it require, and how should one go about implementing it?

## Introduction

In this article we argue that the implementation of electronic laboratory notebooks (eLN) in academic research is overdue, and we provide researchers and their institutions with the background and practical knowledge to select and initiate the establishment of an eLN in their laboratories. Based on our own extensive experience in moving from laboratory notebooks (LN) to eLN, we try to answer the following questions: What does it afford you, what does it require, and how should you go about implementing it?

**Figure 1.**
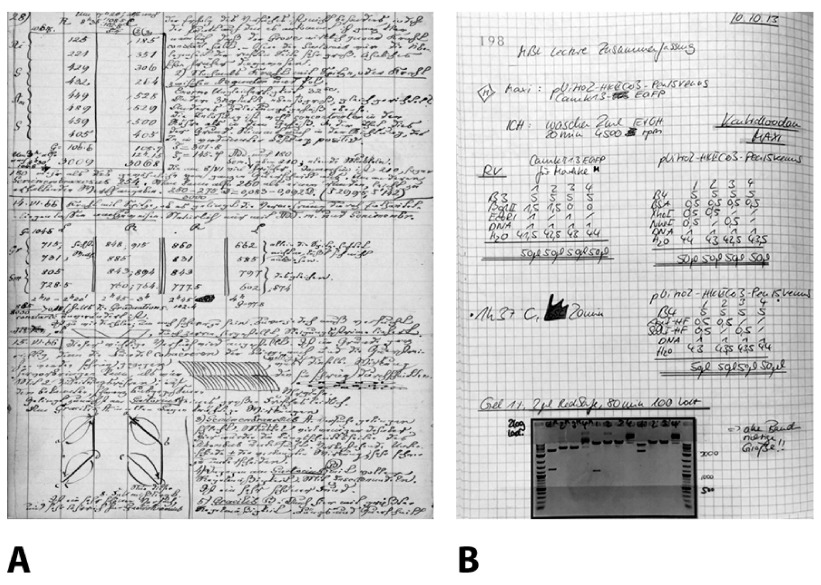
Laboratory notebooks have remained essentially unchanged throughout the last centuries. **A**: Page from the laboratory notebook of the father of experimental electrophysiology, Emil Dubois-Raymond (7 November 1818 – 26 December 1896). [Staatsbibliothek Berlin, 1865–1868, XIII, 22. VII. 65–9. VI. 68, reproduced with permission]. **B**: Pages from a contemporary laboratory notebook from the laboratory of the authors.

Every professional doing active research in the life sciences is required to keep a LN. This is imperative for group leaders, post-docs, students, as well as technicians. LNs are the core element of record keeping, data management, and initial analysis and interpretation of results in research. Details of its specifications, storage, etc. are laid down in institutional, national, as well as international codes of conduct for research integrity and good laboratory practice ^1^. These codes usually stipulate sequentially numbered and bound pages, use of permanent ink, storage for a minimum of 10 years; they often require that entries be signed and dated by a witness. The use of LN has a long history, which parallels the development of modern science since the Renaissance. However, while science has changed dramatically over the last centuries, LNs have remained essentially unchanged since pre-modern science ^2^ ( Figure 1). This is highly remarkable for a number of reasons. For one, most of the data gathered is no longer analog, but digital. Gone are the days when researchers read numbers from instrument for transfer to the LN. Today there is a complex mixture of (often repetitive) protocols, digital images, links to large data files, etc. In addition, the recent realization that there is a ‘reproducibility’ crisis in the life sciences, and an increasing number of high profile cases of research misconduct and subsequent retraction of publications has put record keeping in the spotlight. It is therefore not surprising that the pharmaceutical industry, with its superior resources and regulatory pressures (e.g. Code of Federal Regulations Title 21 ^3^) has moved to eLNs. Many researchers and institutions in academia now realize that the implementation of eLNs is overdue. However, only a tiny fraction of university laboratories are using them. Major hurdles for implementation appear to include ignorance about practical issues, perceived scarcity of available options, and a lack of resources. As part of the implementation of an ISO 9001-certified quality management system, our department (Department of Experimental Neurology) has recently moved from LNs to an eLN. At this department with approximately 100 students, researchers, technicians we carry out multi-professional academic research in preclinical biomedicine with such standard approaches and techniques as *in vivo* and and *in vitro* modeling of disease, cell biology, molecular biology, biochemistry, as well as imaging (from multi-photon microscopy to magnetic resonance imaging). We therefore believe that our experience is applicable to a wide range of research operations in the life sciences.

## Why you will switch to an eLN

We believe that the question is not whether eLNs will become standard or even required in the academic life sciences, but when. The advantages of an eLN are as obvious as the disadvantages of the conventional LN ^4^. Most of the original data obtained in laboratories worldwide is already digital and can easily be integrated or linked to the eLN. eLNs foster collaboration, as protocols, data and concepts can be shared within or between groups. Entries can be time stamped, changes are recorded, versions controlled. Protocols used frequently can simply be integrated as templates. Project progress and eLN use can be easily monitored by group or project leaders. eLNs are searchable, archiving is simple, and copies are easily made for the institution and the individual researcher, many of whom will leave the institution at some point. These features include just a few of the functionalities which are already available in eLNs and are completely absent in a LN. Future eLNs will provide further benefits, including direct data links to standard laboratory hardware through an application programming interface (API) and automatic alerts when instruments are malfunctioning or not calibrated, or direct links to open data repositories (such as Figshare or Dryad). LNs, on the other hand, tend to get lost, must stay within the institution, which in turn has to keep track of them and is charged with keeping reliable records of LNs, storing them and enabling access for at least 10 years.

## Selecting an eLN

If you are contemplating a switch to eLNs, you first need to decide what you expect from it, and match this with your resources (see also below). Table 1 summarizes the principal features of three different categories of eLNs. The simplest form (‘do-it-yourself’ – DIY - type) is a word processor or note-taking system ^5^. It is cheap, easy to use, and has many of the features a conventional LN; its major drawback is its lack of any kind of audit trail or certification. Such DIY-eLNs thus do not even conform to the standards of classical LNs, and therefore are not a serious option for their replacement. Dedicated eLNs have many additional features. Importantly most commercially available eLNs are compliant with the Code of Federal Regulations Title 21 (CFR Title 21) of the US Food and Drug Administration (FDA). CFR Title 21 part 11 sets rigorous specifications for electronic record keeping, including electronic signatures and version control. CFR Title 21 is a must if protection of intellectual property or use of the records for regulatory processes (such as FDA) is a factor ^6^. Dedicated eLNs also allow complex rights management within institutes and workgroups, and can integrate original data. High-end systems include all the features of an eLN, but also function as laboratory information management systems (LIMS), facilitating inventory management or direct link to laboratory equipment (such as microscopes, sequencers, etc.). Not surprisingly, while DIY-eLNs are very easy to use, the increasing functionality of dedicated eLNs and eLNs integrated into LIMS comes at the price of growing complexity in its use. This might be a particular concern when non-academic personnel need to work with the eLN. Another issue is language – the user menus and help functions of practically all commercially available eLNs are in English; only a few allow the user to switch to other languages. Again, this may, in combination with a complex functionality, pose problems, and hamper the acceptance of the eLN in non-academic and less tech-savy work environments. Several articles have reviewed and compared various eLNs ^7^.

**Table 1.** Comparison of specifications of three different types of eLNs. Note that ease of use and the availability as well as power of features of eLNs are inversely related.

Basic systems, such as repurposed word processors (e.g. Word) or note taking systems (e.g. Evernote)	Dedicated, commercial eLNs (e.g. iLabber, Labfolder, eCat)	High end systems (eLNs including a LIMS, e.g. IBDS E-WORKBOOK, iLAB Laboratory Execution System)
Ability to enter text as in conventional handwritten LN	All features from the basic systems plus (see below)	All features from the dedicated, commercial systems plus (see below)
Notes can be made available on multiple devices (stored in the cloud)	Freehand drawing	Inventory management: complete tracking of samples/reagents through all experiments
Attach files to notes	Complex rights management (with roles and individual rights)	Workflows for certain samples, tasks, experiments
Visualization of attachments in the note	21CFR 11 compliance: All previous versions of a note are stored/ changes are logged (full audit trail) Prevents deletion of notes by their author Electronic signatures for completed notes Witnessing and freezing (makes note immutable after the author and a witness have signed it)	Direct link to laboratory equipment (e.g. microscopes, spectrometers, sequencers): Automatic delivery of raw data by device Delivery of metadata (e.g. date of last calibration) from device
Annotation of attachments (e.g. images) *	Extensions/API for customization available	Analysis of raw data within the system
Search within the written text	Assigning of tasks between colleagues *	Data mining (aggregate and cluster structured data)
Search in attachments *	Inventory management: Only amount and location of samples/ reagents	
Notes can be shared with colleagues/ collaborators *		

Abbreviations: API, Application programming interface; 21 CFR 11, code of federal regulations title 21 part 11; LIMS, Laboratory Information Management System. *Indicates that this feature is available in some systems of this category only.

## What you need to get started

For the individual researcher planning to move to an eLN very few requirements exist. Several open source eLNs are freely available (e.g. 8). Some companies offer basic eLN versions for a limited number of users and only as cloud based solutions free of charge (e.g. Labfolder), but for full feature commercial solutions license fees will apply. If a whole workgroup, department, or institution wants to set up an eLN, it gets more complicated. First and foremost, one needs to make sure that the eLN will be accepted by the users. This is not trivial, as many researchers and technicians have been socialized using a conventional LN. They may not be familiar with the many additional useful features provided by an eLN, and are confronted with the challenge and potential distraction of learning how to use a new tool.

To investigate the willingness of staff in a large academic research institution to switch from paper LN to ELN, and to find out what they expect from an ELN, we have surveyed students, technicians, and scientists. We also queried the staff of a research department in the process of switching from LN to ELN. Across professions and career stages the preference was for an intuitive and easy to use interface, a better integration of digital content, use of templates, and the ability to structure notes better. Features considered much less relevant were annotation and freehand drawing, the ability to use mobile devices, or saving time. On an individual level, user expectations and ratings did not substantially change when they progressed from eLN-naive to eLN. More than 70% of those not using an eLN were eager to start working with one, while almost 82% of those already using an eLN now prefer it over the paper version. For details and full results of the surveys, see Supplementary materials 1–4. Although our survey revealed that users of paper laboratory notebooks had a strong motivation for switching to an eLN and a high satisfaction rate for eLNs among those using it, we recommend not to enforce the switch to an eLN. Rather, it should be offered as an opportunity to those who are interested, and scale up its implementation as more group members join in. Sceptics will be able to observe its use, and will very likely want to become users within a short time period.

Another important issue relates to information technology (IT). For workgroups and institutes, the program and data storage will need to run on a server, with local clients, or through a browser interface. Obviously, every lab member using the eLN needs access to a computer. Most eLNs can run on mobile devices and can therefore be taken to the bench or site of experiment even if no computer is present at the site. This requires a wireless connection (WLAN) covering the laboratory or institution. Data can be linked to the eLN by assigning the file and drive name where it is stored. More conveniently, clickable links can directly connect to the data, but this requires that the eLN is physically integrated into the data management structure of the institution. All of this means that in most cases the selection and installation of an eLN from a group level on needs the support of the institutional IT department. They will also be responsible for upgrades, backups, etc. For large-scale installations within whole departments and institutions, training and support contracts need to be considered. Table 2 gives an overview of the requirements.

**Table 2.** Prerequisites for the implementation of an eLN. Note that prerequisites vary with type of eLN and number of users (see Table 1).

Requirement	Comment
Staff willing and able to use the eLN	Note that most systems use the English language for menus and help functions, and user training may be needed for complex LIMS-eLNs
IT infrastructure: Computer access for all users Mobile devices for all users and WLAN Server to host ELN Server to host databases and uploaded data Backup system and load balancer	Only if used on mobile devices Required unless only few individual users use cloud-based system Depends on number of users/volume of data
Personnel for maintenance On-site support User training Administrator of eLN/server Financial License fees or costs for the development custom system Costs for the IT infrastructure (especially storage) Costs for the staff supporting and administrating the eLN	Relevant for larger installations in workgroups and institutions Some commercial systems have basic cloud-based solutions that are free of charge; some systems are unrestricted freeware.

## Obstacles and pitfalls

At present no standard exists for eLNs, and the market is still evolving, so that none of the software makers can guarantee support and further development of their eLN beyond a couple of years. As of now there are no standards for data annotation and integration, therefore migration between different platforms may be difficult or even impossible. In a worst-case scenario (eLN provider goes out of business, no further development or support), the existing eLNs must be saved to pdf-format (including time stamps, addresses linking to stored data etc.), or to html/xml formats, as this will help retain some of the functionality. Such a feature should be mandatory, and is provided by most eLNs. This would essentially mean reverting to a conventional lab book, but the pdf would still provide extra features such as searchability and ease of copying and storage. For eLNs evolving on an open source platform, termination of support of proprietary software is not an issue. However, development or bug fixing of open source software may also be terminated. In addition, such systems may have less support than commercial systems, and support be restricted to tech-savy users or environments with programming capabilities. Another issue relates to the complexity and wealth of functions provided in particular by the high-end eLNs (often part of a LIMS). If using the eLN becomes too complex or restrictive, users may start recording their work outside the eLN. Finally, committing to long-term license fees may be a problem, in particular for individual researchers who may have only fluctuating financial or institutional support.

## Conclusion and recommendations

How biomedical scientists take notes and document their work has not changed much over the last 200 years: They write with a pen in a bound, paginated laboratory notebook. The only major modification is that today, printouts or images of results are often attached ( Figure 1). Data, however, is meanwhile almost exclusively digital, and digital technology provides a plethora of tools for recording, annotating, sharing, processing, and storing all the information that cumulatively drives progress in the life sciences. Scientists use computers for everything and everywhere, privately and professionally, except for documenting their research, experiments, and laboratory procedures. Several reasons may account for the astounding survival of the paper LN. It is a robust and easy to handle ‘technology’, which has been handed down over generations of scientists. At the same time, the emerging eLN market has been dominated by expensive solutions for research and development in large life science companies. Standards for data annotation, exchange or export between different eLN platforms have not yet evolved. There is a hesitation to commit to a specific product that may no longer be supported when the company goes out of business. The inertia of scientists to abandon their cheap and time-honored record keeping system, despite its numerous disadvantages and despite the obvious advantages of electronic solutions, has hampered the development of mature and affordable products for the academic sector. This has led to a vicious circle: Lack of interest on the part of scientists has frustrated the development of dedicated software. Over the last few years, the situation has slowly but substantially changed, and mature and affordable (or even free) eLNs are available. Scientists who overcome their reservation and exchange their LN for an eLN regularly become avid supporters after a short learning period, praising functionalities like group collaboration, use of templates, embedding of data, scheduling, access to the eLN from any computer world-wide, etc. In addition, group leaders and organizations value enhanced documentation and version control, improved supervision of record keeping, as well as backup and archiving of records.

**Box 1. Recommendations**

Ensure willingness of staff to use the novel record keeping technology (ease of use, language of menus).

Clearly define the functionalities that you expect from the eLN. Do not get lost in the almost limitless portfolio of potential functionalities. Remember: You probably just want to replace your paper LN, and not install a new word processor, graphics editor, or groupware system.

Although you may not be aware of it now, you probably want a product which complies to legal requirements like 21CFR11, as well as good scientific practice (full audit trial, restriction on deletion of data, timestamps, ability to freeze and sign entries, among others).

Unless you will be the only user, flexible and hierarchical rights management is very important.

The system needs to be able to tag, filter and search entries. Organization of data in eLN in projects, subprojects, milestones, etc. is a must.

Make sure that all entries, imported data, and links can be exported to a generic format (pdf, zip, xlm, etc.) for backup and reporting as well as allowing a bailout in case the maker of the software stops development, or your funds to pay for licence fees run dry.

Enlist the support of your IT department at an early stage (selection of particular eLN).

Beware of hidden costs (hardware like server, backup devices; on-site support and user training, if applicable etc.)

Besides serving all the obvious functions of a paper LN, eLNs facilitate scientists’ workflow (quick creation and editing of experiments) and collaboration (sharing and reusing information, independent of location, harmonization of work practices). They allow the integration of data, images, files, etc. and can already read data directly from instruments. They eliminate the need to transcribe or cut-paste data from one system to another, thereby avoiding transcription errors. Templates and boilerplate text modules prevent tedious rewriting. eLNs facilitate the retrieval of data or information over long periods of time, improve data quality (legibility), and allow the detailed reconstruction of individual experiments. They facilitate the mobility of researchers. Last, but not least, eLNs promote compliance with Guidelines on Good Laboratory Practice (GLP) and Good Scientific practice, and help intellectual property protection by their compliance to 21CFR11. We have no doubt that eLNs will become standard in most life science laboratories in the near future.

## Supplementary material

**Supplementary material 1.**

Results of an anonymous survey at the Department of Experimental Neurology.

**Supplementary material 2.**

Results of an anonymous survey at Charité Universitätsmedizin Berlin.

**Supplementary material 3.**

Printout of the questions of the survey at the Department of Experimental Neurology.

**Supplementary material 4.**

Printout of the questions of the survey at Charité Universitätsmedizin Berlin.
