# Supplementary material for: A pocket guide to electronic laboratory notebooks in the academic life sciences
Source: F1000Res. 2016 Jan 4;5:2. [Version 1] doi: 10.12688/f1000research.7628.1 (PMC4722687; doi:10.12688/f1000research.7628.1)
Supplement: Supplementary file 1 [file f1000research-5-8214-s0000.tgz › 308e6d57-af08-4b60-9bc6-b4722d5c4e11.docx]

**Supplementary Material 1: Survey 1**

We have surveyed the students, technicians, and scientists of a typical academic research institution (Department of Experimental Neurology at Charite Universitätsmedizin Berlin). While switching from LN to ELN we queried department staff with an anonymous, voluntary questionnaire. We assessed how willing users of classical LNs (n=50) are to switch to an eLN, and which eLN features interest them most. We asked those already using an eLN (n=22) which features were most important to them before they started using the eLN, and how using the eLN has changed this assessment. Across professions and career stages the preference was for an intuitive and easy to use interface, a better integration of digital content, use of templates, and the ability to structure notes better. Features considered much less relevant were annotation and freehand drawing, the ability to use mobile devices, or saving time. On an individual level, user expectations and ratings did not substantially change when they progressed from eLN-naive to eLN. More than 70 % of those not using an eLN where eager to start working with one, while almost 82 % of those already using an eLN now prefer it over the paper version. For a printout of the questions of the survey, see Supplementary Material 3.

To validate the findings of this survey undertaken at the Department of Experimental Neurology in a larger cohort, and to rule out the possibility that the results were biased by the fact that the survey was undertaken during a transition phase, we repeated the survey by inviting the research staff of the entire medical faculty to participate in a similarly structured survey. For results see Supplementary Material 2 and Supplementary Figures 2 and 3.

**Supplementary Figure 1: Scoring of relevance of features by profession.**
