# Supplementary material for: A pocket guide to electronic laboratory notebooks in the academic life sciences
Source: F1000Res. 2016 Jan 4;5:2. [Version 1] doi: 10.12688/f1000research.7628.1 (PMC4722687; doi:10.12688/f1000research.7628.1)
Supplement: Supplementary file 2 [file f1000research-5-8214-s0001.tgz › 028c5ff2-d5c2-4002-b7dc-f7c3a9d09a86.docx]

**Supplementary Material 2:**

To validate the findings of the survey undertaken at the Department of Experimental Neurology (See supplementary material 1) in a larger cohort, and to rule out the possibility that the results were biased by the fact that the survey was undertaken during a transition phase, we repeated the survey by inviting the research staff of the entire medical faculty to participate in a similarly structured survey. This anonymous survey of 214 scientists and technicians from all domains of biomedicine at Charité Universitätsmedizin Berlin asked what hypothetical features they would like to see in an ELN, and which features are less important for them. Participants had to choose 3 items from a list of potential ELN features (left column in Suppl. Figures 2 and 3). Heatmap scale ranks how often a feature was selected. ‘Rank’: #1 most frequently nominated feature, least frequently nominated feature. For a printout of the questions of the survey, see Supplementary Material 3.

Of the 214 respondents, 159 had never used an ELN before (‘naive’), and 42 had some practical exposure (‘user’). Since none of the Charité departments is regularly using an ELN (besides the one we queried for the survey described in supplement 1), it is important to note that this exposure was probably rather superficial and short, that it used freely available software, and was not assisted by any formal training or supervision. This survey basically revealed the same preferences as those collected in an environment of structured transition to an ELN (Supplementary Material 1).

**Supplementary Figure 2: Least relevant features by profession.**

|  | **Group Leader** | **Labmanager** | **Postdoc** | **PhD studentent** | **Undergraduate student** | **Technical Assistant** |
| --- | --- | --- | --- | --- | --- | --- |
| **n naive** | 22 | 9 | 39 | 47 | 8 | 34 |
| **n ELN user** | 3 | 8 | 16 | 7 | 2 | 6 |
|  |  |  |  |  |  |  |
| **share notes and data (naive)** |  |  |  |  |  |  |
| **share notes and data (ELN user)** |  |  |  |  |  |  |
|  |  |  |  |  |  |  |
| **follow progress (naive)** |  |  |  |  |  |  |
| **follow progress (ELN user)** |  |  |  |  |  |  |
|  |  |  |  |  |  |  |
| **intuitive interface (naive)** |  |  |  |  |  |  |
| **intuitive interface (ELN user)** |  |  |  |  |  |  |
|  |  |  |  |  |  |  |
| **mobile devices (naive)** |  |  |  |  |  |  |
| **mobile devices (ELN user)** |  |  |  |  |  |  |
|  |  |  |  |  |  |  |
| **templates (naive)** |  |  |  |  |  |  |
| **templates (ELN user)** |  |  |  |  |  |  |
|  |  |  |  |  |  |  |
| **personal support (naive)** |  |  |  |  |  |  |
| **personal support (ELN user)** |  |  |  |  |  |  |
|  |  |  |  |  |  |  |
| **save time (naive)** |  |  |  |  |  |  |
| **save time (ELN user)** |  |  |  |  |  |  |
|  |  |  |  |  |  |  |
| **better structuring (naive)** |  |  |  |  |  |  |
| **better structuring (ELN user)** |  |  |  |  |  |  |
|  |  |  |  |  |  |  |
| **integration of digital content (naive)** |  |  |  |  |  |  |
| **integration of digital content (ELN user)** |  |  |  |  |  |  |
|  |  |  |  |  |  |  |
| **annotation and freehand drawing (naive)** |  |  |  |  |  |  |
| **annotation and freehand drawing (ELN user)** |  |  |  |  |  |  |

| **Rank** |  |
| --- | --- |
| **1** |  |
| **2** |  |
| **3** |  |
| **4** |  |
| **5** |  |
| **6** |  |
| **7** |  |
| **8** |  |
| **9** |  |
| **10** |  |

**Supplementary Figure 3: Most relevant features by profession.**

|  | **Group Leader** | **Labmanager** | **Postdoc** | **PhD studentent** | **undergraduate student** | **Technical Assistant** |
| --- | --- | --- | --- | --- | --- | --- |
| **n naive** | 22 | 9 | 39 | 47 | 8 | 34 |
| **n ELN user** | 3 | 8 | 16 | 7 | 2 | 6 |
|  |  |  |  |  |  |  |
| **share notes and data (naive)** |  |  |  |  |  |  |
| **share notes and data (ELN user)** |  |  |  |  |  |  |
|  |  |  |  |  |  |  |
| **follow progress (naive)** |  |  |  |  |  |  |
| **follow progress (ELN user)** |  |  |  |  |  |  |
|  |  |  |  |  |  |  |
| **intuitive interface (naive)** |  |  |  |  |  |  |
| **intuitive interface (ELN user)** |  |  |  |  |  |  |
|  |  |  |  |  |  |  |
| **mobile devices (naive)** |  |  |  |  |  |  |
| **mobile devices (ELN user)** |  |  |  |  |  |  |
|  |  |  |  |  |  |  |
| **templates (naive)** |  |  |  |  |  |  |
| **templates (ELN user)** |  |  |  |  |  |  |
|  |  |  |  |  |  |  |
| **personal support (naive)** |  |  |  |  |  |  |
| **personal support (ELN user)** |  |  |  |  |  |  |
|  |  |  |  |  |  |  |
| **save time (naive)** |  |  |  |  |  |  |
| **save time (ELN user)** |  |  |  |  |  |  |
|  |  |  |  |  |  |  |
| **better structuring (naive)** |  |  |  |  |  |  |
| **better structuring (ELN user)** |  |  |  |  |  |  |
|  |  |  |  |  |  |  |
| **integration of digital content (naive)** |  |  |  |  |  |  |
| **integration of digital content (ELN user)** |  |  |  |  |  |  |
|  |  |  |  |  |  |  |
| **annotation and freehand drawing (naive)** |  |  |  |  |  |  |
| **annotation and freehand drawing (ELN user)** |  |  |  |  |  |  |

| **Rank** |  |
| --- | --- |
| **1** |  |
| **2** |  |
| **3** |  |
| **4** |  |
| **5** |  |
| **6** |  |
| **7** |  |
| **8** |  |
| **9** |  |
| **10** |  |

**Supplementary Figure 4: Least relevant features by general computer skills**.

|  | **All** | **Level 1** | **Level 2** | **Level 3** | **Level 4** |
| --- | --- | --- | --- | --- | --- |
| **n naive** | 159 | 3 | 92 | 51 | 13 |
| **n ELN user** | 42 | 1 | 12 | 21 | 8 |
|  |  |  |  |  |  |
| **share notes and data (naive)** |  |  |  |  |  |
| **share notes and data (ELN user)** |  |  |  |  |  |
|  |  |  |  |  |  |
| **follow progress (naive)** |  |  |  |  |  |
| **follow progress (ELN user)** |  |  |  |  |  |
|  |  |  |  |  |  |
| **intuitive interface (naive)** |  |  |  |  |  |
| **intuitive interface (ELN user)** |  |  |  |  |  |
|  |  |  |  |  |  |
| **mobile devices (naive)** |  |  |  |  |  |
| **mobile devices (ELN user)** |  |  |  |  |  |
|  |  |  |  |  |  |
| **templates (naive)** |  |  |  |  |  |
| **templates (ELN user)** |  |  |  |  |  |
|  |  |  |  |  |  |
| **personal support (naive)** |  |  |  |  |  |
| **personal support (ELN user)** |  |  |  |  |  |
|  |  |  |  |  |  |
| **save time (naive)** |  |  |  |  |  |
| **save time (ELN user)** |  |  |  |  |  |
|  |  |  |  |  |  |
| **better structuring (naive)** |  |  |  |  |  |
| **better structuring (ELN user)** |  |  |  |  |  |
|  |  |  |  |  |  |
| **integration of digital content (naive)** |  |  |  |  |  |
| **integration of digital content (ELN user)** |  |  |  |  |  |
|  |  |  |  |  |  |
| **annotation and freehand drawing (naive)** |  |  |  |  |  |
| **annotation and freehand drawing (ELN user)** |  |  |  |  |  |

| **Rank** |  |
| --- | --- |
| **1** |  |
| **2** |  |
| **3** |  |
| **4** |  |
| **5** |  |
| **6** |  |
| **7** |  |
| **8** |  |
| **9** |  |
| **10** |  |

**Level 1** = Beginner (‘No or infrequent use of computers’)

**Level 2** = Moderate (‘Uses word processors and spreadsheets’)

**Level 3** = Intermediate (‘Uses macros and advanced program functions’)

**Level 4** = Advanced (‘Has programming skills’)

**Supplementary Figure 5: Most relevant features by general computer skills (‘level’)**

|  | **All** | **Level 1** | **Level 2** | **Level 3** | **Level 4** |
| --- | --- | --- | --- | --- | --- |
| **n naive** | 159 | 3 | 92 | 51 | 13 |
| **n ELN user** | 42 | 1 | 12 | 21 | 8 |
|  |  |  |  |  |  |
| **share notes and data (naive)** |  |  |  |  |  |
| **share notes and data (ELN user)** |  |  |  |  |  |
|  |  |  |  |  |  |
| **follow progress (naive)** |  |  |  |  |  |
| **follow progress (ELN user)** |  |  |  |  |  |
|  |  |  |  |  |  |
| **intuitive interface (naive)** |  |  |  |  |  |
| **intuitive interface (ELN user)** |  |  |  |  |  |
|  |  |  |  |  |  |
| **mobile devices (naive)** |  |  |  |  |  |
| **mobile devices (ELN user)** |  |  |  |  |  |
|  |  |  |  |  |  |
| **templates (naive)** |  |  |  |  |  |
| **templates (ELN user)** |  |  |  |  |  |
|  |  |  |  |  |  |
| **personal support (naive)** |  |  |  |  |  |
| **personal support (ELN user)** |  |  |  |  |  |
|  |  |  |  |  |  |
| **save time (naive)** |  |  |  |  |  |
| **save time (ELN user)** |  |  |  |  |  |
|  |  |  |  |  |  |
| **better structuring (naive)** |  |  |  |  |  |
| **better structuring (ELN user)** |  |  |  |  |  |
|  |  |  |  |  |  |
| **integration of digital content (naive)** |  |  |  |  |  |
| **integration of digital content (ELN user)** |  |  |  |  |  |
|  |  |  |  |  |  |
| **annotation and freehand drawing (naive)** |  |  |  |  |  |
| **annotation and freehand drawing (ELN user)** |  |  |  |  |  |

| **Rank** |  |
| --- | --- |
| **1** |  |
| **2** |  |
| **3** |  |
| **4** |  |
| **5** |  |
| **6** |  |
| **7** |  |
| **8** |  |
| **9** |  |
| **10** |  |

**Level 1** = Novice

**Level 2** = Moderate (‘Use of word processors and spreadsheets’)

**Level 3** = Intermediate (‘Use of macros and advanced program functions’)

**Level 4** = Advanced (‘Programming skills’)

**Supplementary Figure 6: Least relevant features by familiarity with ELNs**

|  | **ELN naive all** | **Familiar 1** | **Familiar 2** | **Familiar 3** | **Familiar 4** |
| --- | --- | --- | --- | --- | --- |
| **n** | 159 | 40 | 71 | 37 | 11 |
|  |  |  |  |  |  |
| **share notes and data** |  |  |  |  |  |
| **follow progress** |  |  |  |  |  |
| **intuitive interface** |  |  |  |  |  |
| **mobile devices** |  |  |  |  |  |
| **templates** |  |  |  |  |  |
| **personal support** |  |  |  |  |  |
| **save time** |  |  |  |  |  |
| **better structuring** |  |  |  |  |  |
| **integration of digital content** |  |  |  |  |  |
| **annotation and freehand drawing** |  |  |  |  |  |

| **Rank** |  |
| --- | --- |
| **1** |  |
| **2** |  |
| **3** |  |
| **4** |  |
| **5** |  |
| **6** |  |
| **7** |  |
| **8** |  |
| **9** |  |
| **10** |  |

**Familiar 1** = ‘Never heard about ELNs before survey’

**Familiar 2** =’Aware of ELNs, but no personal exposure’

**Familiar 3** = ‘Reflected about ELNs, but no personal exposure’

**Familiar 4** = ‘Reflected about ELNs, witnessed colleagues using ELN’

**Supplementary Figure 7: Most relevant features by familiarity with ELNs**

|  | **ELN naive all** | **Familiar 1** | **Familiar 2** | **Familiar 3** | **Familiar 4** |
| --- | --- | --- | --- | --- | --- |
| **n** | 159 | 40 | 71 | 37 | 11 |
|  |  |  |  |  |  |
| **share notes and data** |  |  |  |  |  |
| **follow progress** |  |  |  |  |  |
| **intuitive interface** |  |  |  |  |  |
| **mobile devices** |  |  |  |  |  |
| **templates** |  |  |  |  |  |
| **personal support** |  |  |  |  |  |
| **save time** |  |  |  |  |  |
| **better structuring** |  |  |  |  |  |
| **integration of digital content** |  |  |  |  |  |
| **annotation and freehand drawing** |  |  |  |  |  |

| **Rank** |  |
| --- | --- |
| **1** |  |
| **2** |  |
| **3** |  |
| **4** |  |
| **5** |  |
| **6** |  |
| **7** |  |
| **8** |  |
| **9** |  |
| **10** |  |

**Familiar 1** = ‘Never heard about ELNs before survey’

**Familiar 2** =’Aware of ELNs, but no personal exposure’

**Familiar 3** = ‘Reflected about ELNs, but no personal exposure’

**Familiar 4** = ‘Reflected about ELNs, witnessed colleagues using ELN’
