# Supplementary material for: A pocket guide to electronic laboratory notebooks in the academic life sciences
Source: F1000Res. 2016 Jan 4;5:2. [Version 1] doi: 10.12688/f1000research.7628.1 (PMC4722687; doi:10.12688/f1000research.7628.1)
Supplement: Supplementary file 3 [file f1000research-5-8214-s0002.tgz › d5e86a11-7757-49be-a357-e5d84c92d1d9.docx]

1) Which of the following profiles describes your position best?

1. Group leader
2. Labmanager
3. Postdoc
4. Postgraduate student (PhD Student)
5. Undergraduate student (labrotation, dr.cand.med., BSc, BSc…)
6. Technical personnel

Comments (if necessary):_____________________________________

2)  Choose the answer which fits best:

1. I have used an ELN, but not on a regular basis and not as the main documentation system of my lab work
2. I use an ELN as the main documentation system of my lab work since
   1. less than three months
   2. between three months and one year
   3. longer than one year

Comments (if necessary):_____________________________________

3) I use computers in my work environment

1. Very often (= many times a day)
2. Often (=every day, but only several times)
3. Some times (=there are days when I do not use a computer)
4. Never

Comments (if necessary):_____________________________________

4) I consider my level in computing

1. Advanced (= I write programs myself)
2. Intermediate (= I sometimes use macros, and advanced program functions)
3. Moderate (= I get around with word processors and spreadsheets)
4. Novice

Comments (if necessary):_____________________________________

5)

The following is a list of potential features of an ELN. Which ones might be very important for you?

Ability to share notes and data of collaborative projects with colleagues

1. Strongly agree
2. Agree
3. Neither agree nor disagree
4. Disagree
5. Strongly disagree

Ability to follow the progress of my whole team on certain projects

1. Strongly agree
2. Agree
3. Neither agree nor disagree
4. Disagree
5. Strongly disagree

Intuitive user interface / easy to lern, easy to use

1. Strongly agree
2. Agree
3. Neither agree nor disagree
4. Disagree
5. Strongly disagree

Ability to use mobile devices (Tablets / Smartphones as input devices for the ELN)

1. Strongly agree
2. Agree
3. Neither agree nor disagree
4. Disagree
5. Strongly disagree

Ability to use templates for recurring types of entries (standard procedure)

1. Strongly agree
2. Agree
3. Neither agree nor disagree
4. Disagree
5. Strongly disagree

A person who gives on site support and supports me in using the system

1. Strongly agree
2. Agree
3. Neither agree nor disagree
4. Disagree
5. Strongly disagree

Saving time in comparison with a paper notebook

1. Strongly agree
2. Agree
3. Neither agree nor disagree
4. Disagree
5. Strongly disagree

Better structuring of notes in comparison with a paper notebook (tagging, sorting by date, and project, searching etc)

1. Strongly agree
2. Agree
3. Neither agree nor disagree
4. Disagree
5. Strongly disagree

Better integration of digital content (pictures, raw data…) in comparison with a paper notebook

1. Strongly agree
2. Agree
3. Neither agree nor disagree
4. Disagree
5. Strongly disagree

Annotation / Freehand drawing inside the ELN

1. Strongly agree
2. Agree
3. Neither agree nor disagree
4. Disagree
5. Strongly disagree

| Feature | Expectation: Before I started using it, I expected that the feature would be very important for me | Satisfaction:  After having used an ELN, the following features were very important to me |
| --- | --- | --- |
| Better integration of digital content (pictures, raw data…) in comparison with a paper notebook | 1. Strongly agree 2. Agree 3. Neither agree nor disagree 4. Disagree 5. Strongly disagree | 1. Strongly agree 2. Agree 3. Neither agree nor disagree 4. Disagree 5. Strongly disagree |
| ..... |  |  |
| ..... |  |  |

6) AN ELN should be rich in features, even at the cost of a more complex interface:

1. Strongly agree
2. Agree
3. Neither agree nor disagree
4. Disagree
5. Strongly disagree

Only ELN users

7) After having used an ELN, I prefer an ELN over a paper notebook

1. Strongly agree
2. Agree
3. Neither agree nor disagree
4. Disagree
5. Strongly disagree

For those who have not used an ELN

7) I would like to try to work with an ELN

1. Strongly agree
2. Agree
3. Neither agree nor disagree
4. Disagree
5. Strongly disagree

8) Comments, if needed

Explain your decision in less then 10 Words. (voluntary)
