# Supplementary material for: A pocket guide to electronic laboratory notebooks in the academic life sciences
Source: F1000Res. 2016 Jan 4;5:2. [Version 1] doi: 10.12688/f1000research.7628.1 (PMC4722687; doi:10.12688/f1000research.7628.1)
Supplement: Supplementary file 4 [file f1000research-5-8214-s0003.tgz › d0f4753a-9ce2-4987-9d43-c0b09a31e167.pdf]

Electronic Lab Notebook Charite

This Survey is about your opinion on electronic lab notebooks, either by the experience you already made, or by the expectations you have.

Welcome

There are 13 questions in this survey

Your Profile

Some questions about you

[]Which of the following profiles describes your position best? *

Please choose **only one** of the following:

- ○ Group Leader
- ○ Labmanager
- ○ Postdoc
- ○ Postgraduate student (PhD Student)
- ○ Undergraduate student (labrotation, dr.cand.med., BSc, BSc…)
- ○ Technical personnel

[]Have you already used an Electronic Labnotebook (ELN) *

Please choose **only one** of the following:

- ○ Yes
- ○ No

[]How familiar are you with the toppic ELN ? *

Only answer this question if the following conditions are met:
Answer was 'No' at question '2 [b]' (Have you already used an Electronic Labnotebook (ELN))

Please choose **only one** of the following:

- ○ Before this survey, I have never heard about the topic ELN
- ○ I know, ELNs exist, but I never had any further direct or indirect contact to this issue
- ○ I have heard and talked about this topic with friends / colleagues but never saw one by myself
- ○ I have in addition to answer 3 seen people using ELNs.

[]For how long do you use an ELN? *

Only answer this question if the following conditions are met:
Answer was 'Yes' at question '2 [b]' (Have you already used an Electronic Labnotebook (ELN))

Please choose **only one** of the following:

- ◯ I have used an ELN, but not on a regular basis and not as the main documentation system of my lab work
- ◯ less than three months
- ◯ between three months and one year
- ◯ longer than one year

[]I use computers in my work environment *

Please choose **only one** of the following:

- ◯ Very often (= many times a day)
- ◯ Often (=every day, but only several times)
- ◯ Some times (=there are days when I do not use a computer)
- ◯ Never

[]I consider my level in computing *

Please choose **only one** of the following:

- ◯ Advanced (= I write programs myself)
- ◯ Intermediate (= I sometimes use macros, and advanced program functions)
- ◯ Moderate (= I get around with word processors and spreadsheets)
- ◯ Novice

[]Additional comment concerning your profile (optional)

Please write your answer here:

Simplicity vs Features

[]

Do you agree to the following statement?

"An ELN should be rich in features, even if this makes the handling less intuitive"

*****

Please choose **only one** of the following:

- Yes
- No

ELN Features: Wich are important?

[]Please choose three Features out of this list which are most important for you (double click or dag and drop)

All your answers must be different.
Please select 3 answers

Please number each box in order of preference from 1 to 10

Please choose at least 3 items.

Please choose no more than 3 items.

- [ ] Ability to share notes and data of collaborative projects with colleagues
- [ ] Ability to follow the progress of my whole team on certain projects
- [ ] Intuitive user interface / easy to lern, easy to use
- [ ] Ability to use mobile devices (Tablets / Smartphones as input devices for the ELN)
- [ ] Ability to use templates for recurring types of entries (standard procedure)
- [ ] A person who gives on site support and supports me in using the system
- [ ] Saving time in comparison with a paper notebook
- [ ] Better structuring of notes in comparison with a paper notebook (tagging, sorting by date, and project, searching etc)
- [ ] Better integration of digital content (pictures, raw data…) in comparison with a paper notebook
- [ ] Annotation / Freehand drawing inside the ELN

ELN Features: Which are not important?

[]Please choose three Features out of this list which are least important for you (double click or dag and drop)

All your answers must be different.
Please select 3 answers

Please number each box in order of preference from 1 to 10

Please choose at least 3 items.

Please choose no more than 3 items.

- [ ] Ability to share notes and data of collaborative projects with colleagues
- [ ] Ability to follow the progress of my whole team on certain projects
- [ ] Intuitive user interface / easy to lern, easy to use
- [ ] Ability to use mobile devices (Tablets / Smartphones as input devices for the ELN)
- [ ] Ability to use templates for recurring types of entries (standard procedure)
- [ ] A person who gives on site support and supports me in using the system
- [ ] Saving time in comparison with a paper notebook
- [ ] Better structuring of notes in comparison with a paper notebook (tagging, sorting by date, and project, searching etc)
- [ ] Better integration of digital content (pictures, raw data…) in comparison with a paper notebook
- [ ] Annotation / Freehand drawing inside the ELN

ELN vs Paper

[]After having used an ELN, I prefer an ELN over a paper notebook *

Only answer this question if the following conditions are met:
Answer was 'Yes' at question '2 [b]' (Have you already used an Electronic Labnotebook (ELN))

Please choose **only one** of the following:

- Yes
- No

[]I would like to try to work with an ELN *

Only answer this question if the following conditions are met:
Answer was 'No' at question '2 [b]' (Have you already used an Electronic Labnotebook (ELN))

Please choose **only one** of the following:

- Yes
- No

Your Comments

If there is anything you would like to let us know, you can write it here

[]

Please write your answer here:

Thank you very much for your help
01.11.2015 – 13:56

Submit your survey.
Thank you for completing this survey.
