# Supplementary material for: A pocket guide to electronic laboratory notebooks in the academic life sciences
Source: F1000Res. 2016 Jan 4;5:2. [Version 1] doi: 10.12688/f1000research.7628.1 (PMC4722687; doi:10.12688/f1000research.7628.1)
Supplement: Peer review history

# Peer review: Referee response for version 1

**DOI:** 10.5256/f1000research.8214.r11788

**Published:** 2016-01-14

**Authors:** Adam Denes

**Affiliations:** 1 MTA Institute of Experimental Medicine, Hungarian Academy of Sciences, Budapest, Hungary

In this article the authors present their views on laboratory record keeping procedures and argue that electronic laboratory notebooks (eLN) would be most suitable to support modern scientific research replacing the traditional, paper based version. One major strength of this paper is the personal experience of the authors with eLN from their own department. As it comes clear from the text, researchers are often reluctant to switch to eLN despite their advantages over the old fashioned laboratory notebooks. The authors arguments for using eLN are well taken: original data generated in laboratories are mostly digital, storage of large files, protocols or images requires electronic data management anyway, whilst data sharing or searching large databases, protocols becomes much easier if using eLN. In addition, eLN run on portable devices and entries can be time stamped and changes recorded, which is required by the scientific community and regulatory pressures. Whilst the authors’ preference is obvious for eLN, they are cautious with their recommendations and clearly outline the potential pitfalls of eLN as well.

 In my opinion, the following issues might deserve a short explanation:

- What is the level of detail researchers should record in eLN? In the case of standard LN, complex data files and large figures  mostly remain electronic, whilst some figures, tables, analysis results and conclusions are incorporated into the LN. However, software running on dedicated machines (flow cytometers, imaging devices, etc) generate large files that are only partially extracted in text, PDF or other forms due to their incompatibility with common word processing tools. Should eLN users attempt to incorporate some of these data into eLN considering limitations in storage space, or such files should remain stored and analysed as earlier?
- How safe do the authors feel keeping most of their research data in an organized manner in eLN using cloud-based systems or large databases that are available from external servers or institutions? Are there measures large research units should consider before switching to eLN to support the protection of intellectual property or their patent applications?

Overall, I find this article very useful and informative. I suspect that in the near future most research labs will not be able to avoid using eLN due to a number of reasons explained clearly in the paper. Practical information from experienced users will greatly support the willingness of researchers to start using eLN and this could also contribute to dealing with reproducibility issues in the future.

I have read this submission. I believe that I have an appropriate level of expertise to confirm that it is of an acceptable scientific standard.

# Peer review: Referee response for version 1

**DOI:** 10.5256/f1000research.8214.r11785

**Published:** 2016-01-08

**Authors:** Barry W. McColl

**Affiliations:** 1 The Roslin Institute , Royal (Dick) School of Veterinary Studies, The University of Edinburgh, Edinburgh, UK

The authors present a persuasive argument for replacing conventional lab notebooks with electronic versions (eLNs). Crucially, they outline the current drawbacks of conventional lab books in the current age and why there is a need to consider alternatives. One comment would be that the authors could perhaps have indicated if alternatives other than the systems mentioned in the article exist?

The benefits of adopting eLNs described seem clear and comprehensive but importantly the potential weak points are also considered to give some balance. A similarly balanced tone is evident in discussion of the willingness of researchers to adopt eLNs and their reaction to doing so. Thus while the article is clearly presenting the authors' favoured opinion they also reflect on alternative views.

As commented on by the authors, adopting an electronic system particularly in larger departments or organisations will need the appropriate IT infrastructure and support. While the authors surveyed opinions from researchers it might also have been informative to gauge and record opinion from IT professionals as to some of the practical issues of particular relevance to lab personnel.

Overall, this article is very well structured and presents a helpful and informative perspective and primer on this emerging issue.

I have read this submission. I believe that I have an appropriate level of expertise to confirm that it is of an acceptable scientific standard.

# Peer review: Referee response for version 1

**DOI:** 10.5256/f1000research.8214.r11789

**Published:** 2016-01-06

**Authors:** Christoph Kleinschnitz, Eva Geuß

**Affiliations:** 1 Department of Neurology, University of Würzburg, Würzburg, Germany; 2 University Hospital Würzburg, Würzburg, Germany

Dirnagl and Przesdzing provide a highly innovative and comprehensive summary for the use of electronic laboratory notebooks (eLNs) in life science research. They highlight advantages of eLNs over traditional hand written LNs, comment on possible obstacles and inform readers about distinct features/specification of different types of eLN systems, e.g. complex rights management, integration of original data, direct link to laboratory equipment etc. In addition, they offer a step-by-step instruction of how to establish eLN systems in everyday laboratory practice based on the successful implementation of eLNs in their own institution. From an internal survey of their staff (scientist, students, technicians) they deduce which features of eLN systems were considered most desirable and which ones were regarded less relevant.

In total, the article is highly informative for any researcher working in the life science field and is a very well suited guide for everybody who plans to switch from LN to eLN.

We have read this submission. We believe that we have an appropriate level of expertise to confirm that it is of an acceptable scientific standard.

# Peer review: Referee response for version 1

**DOI:** 10.5256/f1000research.8214.r11787

**Published:** 2016-01-05

**Authors:** Thomas A. Kent

**Affiliations:** 1 Department of Neurology, Stroke Outcomes Laboratory, Baylor College of Medicine, Houston, TX, USA

This is a valuable description of the author’s experience with electronic laboratory notebook implementation within their department. There can be much value in educating the broader scientific community with respect to both their experience, the experience of those they surveyed and the available options for the researcher.

The manuscript can be improved through better definition of terms and procedures related to the eLN with which the average laboratory scientist may not be familiar. Some examples follow:

The title is appropriate and the abstract is a good summary.

Introduction:

The authors department pursued ISO 9001-certified laboratory quality management system. Could they expound on what this means, why they chose to pursue it and what it entails? What kind of resources did they devote to this and what did they hope to gain in return?

“Why you will switch to an eLN”

While suitably provocative, perhaps reword the section title as “Why you will switch to an eLN” seems to put the answer before the research is presented. Perhaps a more neutral statement might not appear that the conclusion has been pre-judged.

In this section, the authors list several advantages to an eLN, including for example, that original data can be “easily integrated or linked to the eLN”. I assumed this meant a direct link to the eLN from the laboratory equipment, but later in this paragraph, the authors describe such a direct link as in the future. Could they then explain how data is easily integrated in the eLN? This question may be a major one for those used to using written notebooks where printouts are often physically attached. And if the data is not directly loaded into the eLN, how is it searchable? Are they scanning data through OCL or some other method? Some details would help the reader visualize how this system might help them.

“What you need to get started”

This is a well written section with a nice summary Table of the different options. If possible, it might be beneficial to discuss costs, perhaps within an order of magnitude for the different systems, although not if this is proprietary information. 

With respect to the surveys, while the heat maps are useful, it is not easy to follow whether there is a pattern to the different features. Perhaps an analysis using the highest/lowest scores graphically might be more helpful.  It does not appear that any pitfalls were included in the questions. In the article, the authors later discuss possibility of loss of technical support, updates and possibility that the sponsor may go out of business. It is not clear that the survey included that possibility and how the respondents might have answered.  I would consider this a modest weakness, but would like to hear some comment from the authors.

“Obstacles and pitfalls”

This is an excellent section that is most useful to the potential purchaser of a system and what are the minimum requirements that should be available should the support be terminated.

This is an important section. Are there alternatives? For example, upfront costs and uncertain future of any wholly integrated platform are reasons that some labs have set up their own cloud based systems, although without all the functionality of an integrated system and without FDA certification, but the data can be easily referenced to physical lab books and as such provide some of the advantages of a whole integrated system.

I have a question for the authors: Do they think that “electronic laboratory notebook manager” may become a new, sought after, position analogous to the “network manager” that is needed even though there are multiple commercial products available and standards widely accepted? Perhaps the pharma experience on how to manage a large, integrated eNL might be helpful here if the authors are aware of them, or perhaps they could describe how at the department whether the management of the eLN is the responsibility of specific individuals.

“Conclusions and recommendations”

This is a good summary of the authors’ thoughts.  Perhaps a little harsh with the term “inertia of scientists”, there may be many reasons that scientists have not embraced eLN in their work, particularly if it is a small laboratory or one strapped for resources other than inertia.

The authors only once mention the important issue of need for restrictions on alteration of data, and only in the Box 1.  Perhaps some mention of this issue would be helpful in the text as well.

The final list of recommendations is an excellent primer for exploring the possibility of an eLN.

I commend the authors for their efforts to increase the visibility of such systems and providing their experience and perspective on this topic.

I have read this submission. I believe that I have an appropriate level of expertise to confirm that it is of an acceptable scientific standard.
